# Emerging Role of *Helicobacter pylori* in the Immune Evasion Mechanism of Gastric Cancer: An Insight Into Tumor Microenvironment-Pathogen Interaction

**DOI:** 10.3389/fonc.2022.862462

**Published:** 2022-06-20

**Authors:** Zhifang Li, Wenqing Zhang, Jinyang Bai, Jing Li, Hong Li

**Affiliations:** ^1^Shanxi Medical University, Taiyuan, China; ^2^The Second Hospital of Shanxi Medical University, Taiyuan, China; ^3^Shanxi Traditional Chinese Medicine Hospital, Taiyuan, China

**Keywords:** gastric cancer, *Helicobacter pylori*, tumor microenvironment, immune evasion, stromal cells

## Abstract

*Helicobacter pylori* (*H. pylori*) infection is the strongest causative factor of gastric cancer. Growing evidence suggests that the complex crosstalk of *H. pylori* and the tumor microenvironment (TME) exerts a profound influence on gastric cancer progression. Hence, there is emerging interest to in-depth comprehension of the mechanisms of interplay between *H. pylori* and the TME. This review discusses the regulatory mechanisms underlying the crosstalk between *H. pylori* infection and immune and stromal cells, including tumor-associated macrophages (TAMs), neutrophils, dendritic cells, myeloid-derived suppressor cells (MDSCs), natural killer (NK) cells, B and T cells, cancer associated fibroblasts (CAFs), and mesenchymal stem cells (MSCs), within the TME. Such knowledge will deepen the understanding about the roles of *H. pylori* in the immune evasion mechanism in gastric cancer and contribute to the development of more effective treatment regimens against *H. pylori*-induced gastric cancer.

## Introduction

Gastric cancer is a global health issue, with over 1 million newly diagnosed cases globally each year (1). Despite the descended morbidity and mortality of this malignancy during the last five years, it is still the third major cause of cancer-related deaths (2). The prognosis of advanced gastric cancer is undesirable with a five-year survival rate of less than 30%. Platinum-fluoropyrimidine combination chemotherapy represents the first-line therapeutic option against advanced patients (1). However, patients usually have a low complete response rate to chemotherapy, with substantial toxicity. Gastric cancer is diagnosed histologically following endoscopic biopsy as well as staged utilizing computed tomography, endoscopic ultrasound, positron emission tomography, and laparoscopy. Currently, histopathological classification schemes designate gastric cancer as intestinal or diffuse in accordance with the morphology, differentiation, and cohesion of glandular cells. Intestinal gastric cancer is preceded through alterations in the gastric mucosa as the Correa cascade that evolves *via* inflammatory response, metaplasia, dysplasia, or adenocarcinoma. Diffuse gastric cancer lacks cell adhesion and exhibits a diffuse aggressive growth feature. Recently, comprehensive evaluation of the Cancer Genome Atlas (TCGA) has categorized this malignancy as 4 molecular subtypes: genome stable (GS), microsatellite instability (MSI), EBV infection, as well as chromosomal instability (CIN) (3). Moreover, the Asian Cancer Research Group (ACRG) project offers a new molecular classification, including MSI subtype, microsatellite stable with epithelial to mesenchymal transition (MSS/EMT), MSS/TP53 mutant (MSS/TP53+), and MSS/TP53 wild-type (MSS/TP53-) subtypes (4). In terms of histopathological or molecular classifications, gastric cancer is not an isolated mass of cancerous epithelial cells. In contrast, these tumors present a complex morphology in which tumor cells are surrounded by the tumor microenvironment (TME) (5). The TME contains extracellular matrix (ECM), stromal cells, immune cells, and secreted factors, which present high correlations to gastric cancer progression and therapeutic responses (6). The innate immune cell populations (macrophage, neutrophil, dendritic cell, innate lymphoid cell, myeloid-derived suppressor cell, natural killer cell) as well as adaptive immune cell populations (T cell and B cell) trigger gastric cancer progression within the TME (7). Innate and adaptive immune responses exert critical functions in tumor immune surveillance as well as suppression of tumor progression. Tumor cells inhibit the immune system, and thus evade immune disruption and facilitate tumor growth, and metastases. Tumor immune surveillance is capable of recognizing and eliminating tumor cells. Tumor immune escape permits tumor cells to proliferation and metastases following escaping immune surveillance and thus results in unfavorable clinical outcomes. Numerus factors participate in the process of tumor immune escape, especially *Helicobacter pylori* (*H. pylori*) infection.

*H. pylori* is a gram-negative microaerobic spiral rod-shaped bacteria that colonizes the surface of human stomach mucosa (2). It is a highly invasive microorganism and is one of the reasons for the highest incidence of chronic infections in the world, though more than 80% of infected patients are still asymptomatic (2). At present, several virulence-related genes in the genome of *H. pylori* have been confirmed, mainly cytotoxin-associated gene A (cagA), vacuum toxin gene A (vacA), induced by contact epithelium (iceA), blood group antigen-binding Adhesin gene (babA), etc. (2). Over 50% of individuals are infected with *H. pylori* across the globe. Nearly all cases of gastric cancer are linked with *H. pylori* infection (8). *H. pylori* eradication is capable of preventing gastric cancer progression and quadruple therapy (bismuth quadruple and concomitant) is the recommended first-line therapeutic option (8). However, antibiotic resistance with a growing prevalence is the main reason for the therapeutic failure of *H. pylori*. Growing evidence suggests the crucial roles of *H. pylori* infection in immune evasion mechanisms during gastric cancer progression (8). The persistence of *H. pylori* infection results in an immunosuppressive microenvironment as well as allows gastric carcinoma cells to evade immune surveillance (8). In this review, we summarize a comprehensive outline of research advances of the molecular mechanisms concerning the crosstalk of *H. pylori* with components within the TME of gastric cancer, suggesting the crucial roles of *H. pylori* infection in tumor immune escape.

## The Crosstalk of *H. pylori* With Innate Immune Cells Within the Tumor Microenvironment

### Tumor-Associated Macrophages

Within the TME, macrophages, known as TAMs, are the most abundant immune cells. TAMs are categorized as two subtypes: M1 and M2 (9). M1 macrophages (also called as classical macrophages) can be primarily stimulated by IFN-γ, TNF-α, and LPS; M2 macrophages can be induced by IL-4 (9). The polarization and recruitment of macrophages produce pro-inflammatory and pro-cancerogenic cytokines and thus sustain the initiation and progression of gastric carcinoma (9). *H. pylori*-induced chronic inflammation exerts a crucial regulatory role in gastric carcinogenesis and TAMs involved in this process. The polarization and recruitment of macrophages supply pro-inflammatory and pro-tumorigenic cytokines and thus support the development and progression of gastric cancer (9). The degree of *H. pylori* infection is linked with macrophage polarization by interplay of reactive oxygen species (ROS) with hypoxia inducible factor 1 subunit α (HIF-1α) (10). HIF-1α can directly or indirectly trigger tumor development *via* modulating immune surveillance escape through inducing immunosuppressive factors and downstream targets. Inhibition of macrophage polarization can ameliorate *H. pylori*-induced gastric injury (11). Exosomes are small extracellular vesicles, which are critical mediators of cell-cell communication. *H. pylori* infection triggers the up-regulation of mesenchymal-epithelial transition factor (MET) in exosomes and activates tumor-associated macrophages through IL-1β, thereby promoting gastric cancer progression (12). *H. pylori* triggers nitric oxide (NO) release in macrophages that leads to methylation of runx3 in gastric epithelial cells (13). Runx3 methylation is linked with differentiation, nodal metastases, and unfavorable clinical outcomes of gastric cancer. Experimental evidence shows that suppression of runx3 triggers gastric carcinoma progression as well as metastases. *H. pylori* also promotes the release of TNF-α from macrophages and thus up-regulates the expression of C-X-C motif chemokine receptor 4 (CXCR4) in gastric cancer cells (14). CXCR4 is a seven-span transmembrane G-protein coupled receptor, which is the main receptor of CXCL12. CXCL12 is released from stromal cells within the TME and thus binds to CXCR4 on the surface of tumor cells, eventually promoting tumor metastases and unfavorable survival outcomes. Notch signaling can facilitate the activation and bactericidal activities of macrophages, and one of its ligands Jagged1 enhances macrophage-mediated response to *H. pylori* (15). CXCL12/CXCR4 signaling-mediated macrophage polarization can trigger gastric cancer metastases (16). MiR-22 inhibits gastric cancer cell growth through triggering a deficiency in endogenous S-adenosylmethionine, which can sustain NLRP3 expression and attenuate *H. pylori*-induced gastric cancer initiation (17). *H. pylori* infection weakens miR-22 expression and thus up-regulates NLRP3 inflammasome activation and release of oncogenic mature IL-1β, thereby triggering uncontrolled proliferation of epithelial cells and the occurrence of gastric cancer (8).

### Neutrophils

Neutrophils are the most abundant circulating leukocytes in cancer patients, which present two forms: circulating neutrophils that circulate freely and are recruited into tumors as well as peripheral neutrophils that bind to the capillary endothelium (18). Persisting and increasing neutrophil infiltrations are linked with gastric cancer progression (18). Hepatoma-derived growth factor (HDGF) displays high expression in gastric carcinoma tissues, which is linked with lymph node metastases and undesirable clinical outcomes. Additionally, the differentiation of mesenchymal stem cells into myofibroblast-like cells induced by HDGF triggers the progression of *H. pylori*-induced gastric cancer. Recent research has proposed that HDGF expression is up-regulated both in tumors and peripheral blood of *H. pylori*-infected patients, which mediates *H. pylori*-triggered neutrophil recruitment or activation of inflammatory TNF-α/COX-2 pathway, and thus promotes gastric carcinogenesis (19).

### Dendritic Cells

Dendritic cells are professional antigen-presenting cell populations, which can induce antigen-specific adaptive immune response that is of importance for immune surveillance and tolerance (20). Impaired function of dendritic cells results in the ineffective innate and adaptive immune responses against *H. pylori* for gastric cancer populations (20). *H. pylori* suppresses the maturation of dendritic cells through IL-10-independent activation of the signal transducer and activator of transcription 3 (STAT3) signaling, potentially favoring chronic infection, and promoting gastric carcinogenesis (21). *H. pylori*-induced immune evasion is mediated by dendritic cell-induced Th17/Treg balance towards Treg-biased responses and inhibition of Th17 immunity (22). MiR-375 expression is frequently decreased in gastric carcinoma as well as weakens cell proliferation through targeting JAK2 oncogene. *H. pylori* infection downregulates its expression in gastric cancer cells and promotes the release of cytokines IL-6, IL-10, and VEGF *via* the JAK2-STAT3 signaling, and thus induces the maturation of dendritic cells and the reduction in the number of CD4+ and CD8+ T cells (23).

### Myeloid-Derived Suppressor Cells

MDSCs are a heterogeneous subset of immature myeloid cell populations with immunosuppressive function. MDSCs have been considered as a main obstacle of immunotherapy (24). Numerous scientists are exploring the inhibitory products against MDSCs and exploiting novel therapies that may enhance the efficacy of immunotherapies. Although immunotherapies mainly focus on the manipulation of T cells, targeting MDSCs provides another insight for anti-cancer therapy. The differentiation and function of MDSCs may be mediated by *H. pylori* infection. CXCL8 is a crucial inflammatory chemokine induced by *H. pylori* infection. This chemokine is up-regulated in gastric cancer and is linked with an undesirable survival outcome and tumor metastases (24). CXCL8 can promote the recruitment of MDSCs to tumors *via* binding CXCR1/2. MDSCs restrain the antitumor immune response primarily through weakening T cell function. Kruppel-like factor 4 (KLF4) is an underlying tumor suppressor in gastric carcinoma. *H. pylori* infection results in KLF4 inactivation in gastric carcinoma with a Tet Methylcytosine Dioxygenase 1 (TET1)-independent DNA methylation mechanism (25). *H. pylori* infection up-regulates CXCL8 expression through down-regulating KLF4 expression in gastric cancer cells and thus facilitates the recruitment of MDSCs, thereby shaping an immunosuppressive microenvironment (26). Hence, effective inhibition and blockade of CXCL8 and disruption of the immunosuppressive microenvironment are of importance for improving the effects of immunotherapy in gastric cancer. *H. pylori*-induced programmed death ligand-1 (PD-L1) expression within the gastric epithelium is mediated through the Hedgehog (Hh) pathway that is a contributor of *H. pylori*-induced atrophic gastritis progressing to gastric cancer. MDSCs require the activation of the Hh pathway-mediated transcription factor GLI1 and thus promotes neoplastic transformation (27). The myeloid differentiation factor Schlafen4 (Slfn4) represents a subpopulation of MDSCs in the gastric with *H. pylori*-induced spasmolytic polypeptide-expressing metaplasia (SPEM). MiR-130b is required for the T-cell suppressor phenotype displayed by the SLFN4-positive cells, which promotes *H. pylori*-induced gastric carcinogenesis (28). Depletion of MDSCs can sensitize gastric cancer cells to anti-programmed death-1 (PD-1)/PD-L1 agents (29).

### Natural Killer Cells

NK cells, large granular innate lymphoid cells, are a main contributor to immunosurveillance as well as control of tumor progression through mediating apoptosis of gastric cancer cells (30). NK cells mainly mediate cytotoxic resistance through two mechanisms: cancer cells escape NK cell-induced response through co-suppressive signals, resulting in anergic or irresponsiveness states of the immune subpopulation, and cancer cells escape NK cell effector activities following recognition of target cells (such as ineffective perforin binding) (30). High NK-cell infiltration is an indicator of more favorable clinical outcomes of gastric carcinoma subjects, constituting the first line of defense against cancers. *H. pylori* infection alters NK cell function within the TME. NK cells in the peripheral blood of gastric cancer patients possess a serious suppression capacity in producing IFN-γ following *H. pylori* infection (30). IFN-γ is an important cytokine secreted from NK and NK T cells or activated T cells within the TME. Innate and adaptive antitumor immune responses can trigger the secretion of IFN-γ. Oppositely, IFN-γ induces feedback suppression, thereby compromising antitumor immune responses. Moreover, IFN-γ upregulates the expression of immune suppressive factors within the TME. Especially, IFN-γ can activate the PD-1 pathway *via* directly upregulating PD-L1/2 in cancer, immune as well as stromal cell populations, and thus interacts with PD-1 on T cells, thereby downregulating the cytotoxic responses. NK cells can kill susceptible target tumor cells with a perforin-dependent mechanism. Perforin is a pore-forming protein expressed only in killer cells, allowing cytotoxic proteases (31). *H. pylori* down-regulates perforin production in gastric cancer cells-co-cultured CD56+ NK cells, indicating the decrease in killing efficiency of NK cells (32).

## The Crosstalk of *H. pylori* With Adaptive Immune Cells Within the Tumor Microenvironment

B and T cells are central mediators of adaptive immunity (33). *H. pylori* infection deregulates T and B cells to mediate immune escape of gastric epithelial cells (34). Immune score has been exploited for estimating the adaptive immune compositions within the TME. In accordance with immune score, tumors are categorized into four subtypes: hot, altered-excluded, altered-immunosuppressed, as well as cold (35). Hot tumors present the features of enhanced infiltrations of PD-1- or CTLA4-expressing cytotoxic T lymphocytes as well as tumor cells expressing costimulatory molecules that are capable of maintaining T-cell functions (36). Intriguingly, hot tumors present the features of the presence of local inflammatory responses as well as high responses to immunotherapy. T-cell exclusion inside the tumors, triggered by the presence of abnormal vasculature and fibrotic nets, represents a major characteristic of altered-excluded immune tumors. Altered-immunosuppressed tumors present the intermediate infiltrations of exhausted T cells and the increased density of soluble inhibitory factors and immune-suppressive cell populations. Cold tumors present the characteristics of the absence of T-cell infiltrations because the cells or mechanisms underlying T-cell priming or activation are lacking. *H. pylori*-induced adrenomedullin facilitates IFN-γ-producing T-cell responses within the gastric microenvironment (37). The production of suppressive cytokine IL-10 in *H. pylori*-infected gastric cancer patients is elevated and thus results in a decreased cytotoxic anti-tumor T-cell response in the gastric mucosa, thereby contributing to gastric carcinogenesis (38). *H. pylori* VacA targets myeloid cells in the gastric mucosa and thus creates a tolerogenic environment that promotes regulatory T-cell (Treg) differentiation and suppresses effector T-cell priming and functionality (39).

**Figure 1:**
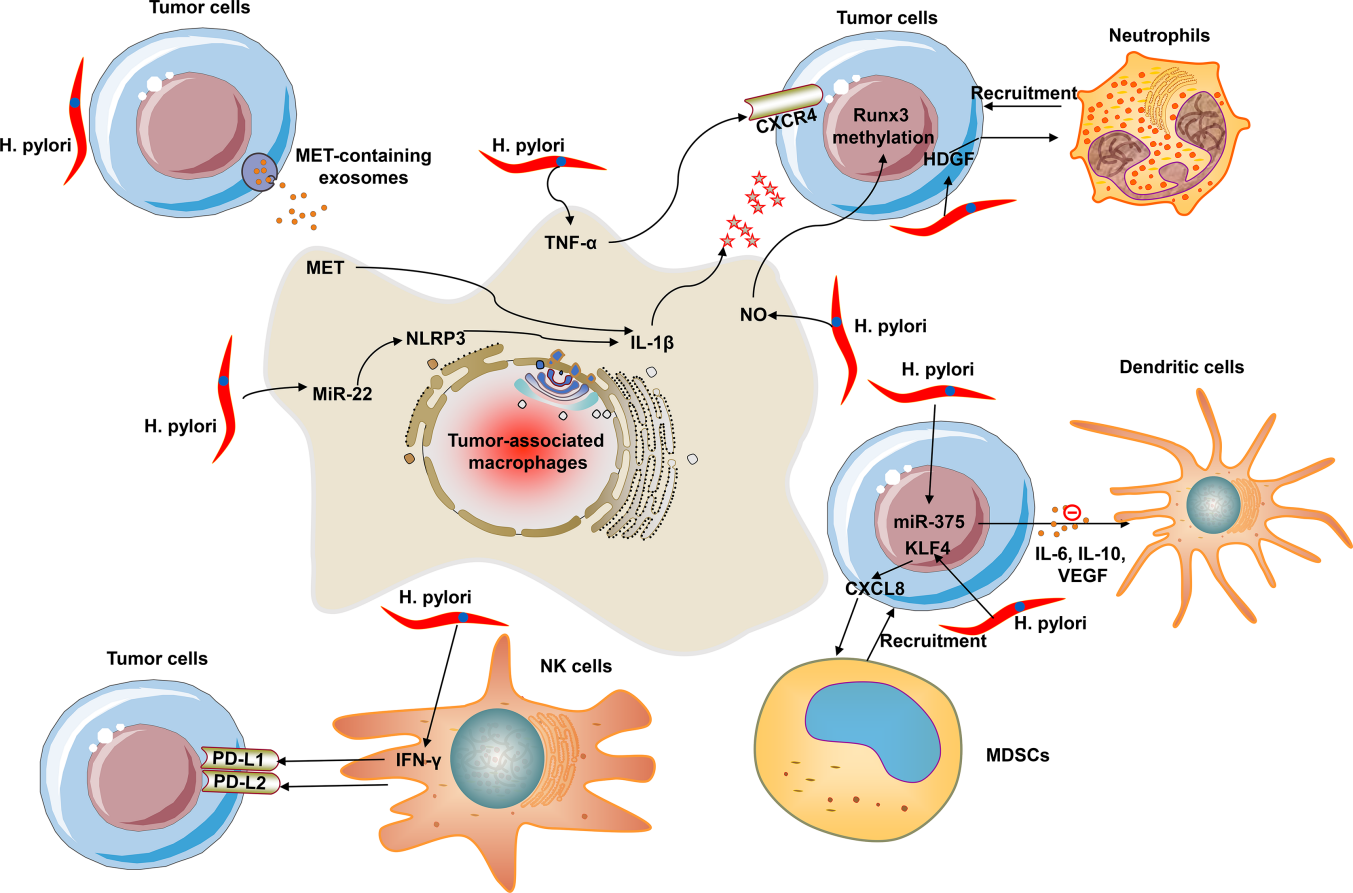
The crosstalk of H. pylori with innate immune cells within the tumor microenvironment (TME). Innate immune cells mainly contain tumor-associated macrophages (TAMs), neutrophils, dendritic cells, myeloid-derived suppressor cells (MDSCs), and natural killer (NK) cells.

**Figure 1** summarizes the crosstalk of *H. pylori* with innate immune cells within the TME, including TAMs, neutrophils, dendritic cells, MDSCs, and NK cells.

## The Crosstalk of *H. pylori* With Cancer Associated Fibroblasts Within the Tumor Microenvironment

CAFs are a major stromal component that display massive infiltrations within the TME (40). CAFs are mainly distributed around blood vessels or in the fibrous interstitium around tumors, secreting cytokines, ECM components, and related enzyme molecules (41). ECM provides physical support for cells in the TME and plays an important role in cell adhesion and infiltration. ECM deposition can produce dense fibrous interstitium that envelops tumors, which makes tumor tissues more brittle and firmer than normal tissues, thereby forming a physical barrier that hinders immune cell infiltration, and inhibiting anti-tumor drugs from targeting the TME (40). Moreover, the matrix metalloproteinases secreted by CAFs can reshape ECM, release chemokines, growth factors, and pro-angiogenesis factors, and promote the malignant transformation of tumors. With the rapid growth of tumors and vascular alienation, insufficient blood supply often occurs in the tumors and long-term hypoxia (42). Meanwhile, cell metabolism will increase the accumulation of lactose and hydrogen ions to form the acidic TME. The activation of a cascade of signals caused by vascular defects and metabolic disorders promotes the formation of immunosuppressive TME (43). CAF mainly affects TME through four perspectives: tumor cell proliferation and metastases, angiogenesis, ECM remodeling, and immune inhibition. *H. pylori* can activate gastric fibroblasts into cells possessing CAFs. *H. pylori* infection increases vascular adhesion molecule 1 (VCAM1) expression in CAFs within the TME through activating the JAK/STAT1 pathway, and CAF-derived VCAM1 interacts with integrin αvβ1/5 in gastric cancer cells to trigger cancer invasion (40). *H. pylori* infection induces the cyclooxygenase-2 (COX-2)/prostaglandin E2 (PGE2) pathway and enhances PGE2 production, leading to the hypermethylation of miR-149 in CAFs as well as the increase in IL-6 secretion. Epigenetic silencing of miR-149 in CAFs mediates the interplay of CAFs with gastric cancer cells within the TME (44).

## The Crosstalk of *H. pylori* With Mesenchymal Stem Cells Within the Tumor Microenvironment

Epithelial–mesenchymal transition (EMT) process may confer mesenchymal phenotype and characteristics to epithelial cells (45, 46), with the abnormality of epithelial polarization and cell-to-cell junction as well as acquiring mesenchymal and motile phenotypic changes (47). *H. pylori* infection triggers the EMT of epithelial cell populations in the stomach mucosa. Moreover, this process can result in the appearance of CSC characteristics in gastric cancer. CSCs are a rare cell subset in tumors, which can initiate the progression and spread of tumors to induce distant metastasis. *H. pylori* infection can unveil CSC-like properties through inducing EMT-like alterations in gastric epithelial cells *via* CagA (47). Hippo pathway effectors yes-associated protein (YAP) along with transcriptional co-activator with PDZ binding motif (TAZ) mediates gastric carcinoma occurrence or development. TAZ activation responding to *H. pylori* can result in *H. pylori*-triggered EMT as well as CSC features. Thus, TAZ up-regulation constitutes a contributor of early conversion during *H. pylori*-induced gastric cancer initiation (48). Bone marrow-derived MSCs facilitate *H. pylori*-induced gastric cancer progression *via* secretion of thrombospondin-2 (49).

## Discussion

*H. pylori* infection has been considered as a microorganism that is highly effective in triggering inflammatory response within the stomach. Recent research has revealed a synergistic interplay of *H. pylori* infection with the components within the TME. An in-depth comprehension of how *H. pylori* and these cell populations interact can provide novel ideas and perspectives in treating gastric cancer and promising biomarkers for early detection. As mentioned, the interactions of *H. pylori* infection with TAMs, neutrophils, dendritic cells, MDSCs, NK cells, B and T cells, CAFs, and MSCs exert crucial roles in gastric carcinogenesis.

## Future Perspectives

Because *H. pylori* infection can induce strong immune response in the stomach, the resulting inflammation can facilitate gastric cancer progression. Most studies investigating the influence of *H. pylori* infection on the mentioned cell populations within the TME have been conducted *in vitro*. Furthermore, the exact mechanisms underlying the interplay of *H. pylori* infection with the TME should be addressed, assisting better expounding of the roles of *H. pylori* infection in gastric carcinogenesis. More biomedical or pharmaceutical regimens specifically targeting *H. pylori* infection can offer potential treatment methods for gastric cancer prevention.

## Author Contributions

All authors made substantial contributions to conception and design, acquisition of data, or analysis and interpretation of data; took part in drafting the article or revising it critically for important intellectual content; gave final approval of the version to be published; and agree to be accountable for all aspects of the work.

## Funding

This work was funded by Shanxi Provincial Health Commission Project (RK-26); Shanxi Provincial Science and Technology Department Project (2018041056-6); The Second Hospital of Shanxi Medical University Fund Project (201702-2); Research on the current situation and countermeasures of the transformation of scientific and technological achievements in large general hospitals (202104031402141); Research on the implementation of medical alliance management model in public hospitals and primary medical and health service institutions (202104031402138).

## Conflict of Interest

The authors declare that the research was conducted in the absence of any commercial or financial relationships that could be construed as a potential conflict of interest.

## Publisher’s Note

All claims expressed in this article are solely those of the authors and do not necessarily represent those of their affiliated organizations, or those of the publisher, the editors and the reviewers. Any product that may be evaluated in this article, or claim that may be made by its manufacturer, is not guaranteed or endorsed by the publisher.

## Abbreviations

TME, tumor microenvironment; ECM, extracellular matrix; *H. pylori*, *Helicobacter pylori*; TAMs, tumor-associated macrophages; NK, natural killer; CAFs, cancer associated fibroblasts; EMT, epithelial–mesenchymal transition.
